# The BLUES database: A health disparities registry for pregnant mothers

**DOI:** 10.1186/1471-2105-14-S17-A6

**Published:** 2013-10-22

**Authors:** Somchan Vuthipadadon, Mark Sakauye, Emanuel Villa, Emin Kuscu, Netasha Bowers, Teresa Franklin, Linda Moses-Simmons, Ian M Brooks

**Affiliations:** 1 Office of Biomedical Informatics, The University of Tennessee Health Science Center, Memphis, TN 38163, USA; 2 Department of Obstetrics and Gynecology, The University of Tennessee Health Science Center, Memphis, TN 38163, USA

## Background

For more than a decade, Tennessee has been consecutively ranked nationally in the top 5 for Infant Mortality Rate (IMR), and in 2012 ranked 3rd with an IMR of 8.3 where the national rate is at 5.98. Some of the primary risk factors for IMR include low birth weight, education, social support, prenatal care, nutrition and exercise, and effective use of community resources, services. An underlying racial and economic component can be found within these statistics. Within Tennessee, Hamilton and Shelby counties have African American IMRs that are two and three times that of whites.

## Materials and methods

To begin to address these health disparities, the Blue Cross Blue Shield Foundation of Tennessee has partnered with UTHSC to initiate the BLUES Project: Building Lasting Unshakeable Expectations into Successes. BLUES is an intensive community outreach, education and research project that focuses on reducing risk factors for low birth weight and related infant mortality rates. Through group and individual training sessions the project aims to empower participants to establish and achieve attainable life goals. These sessions include topics of particular relevance to African American women in urban communities, such as pregnancy planning, birth control, financial planning, substance abuse and domestic abuse mitigation. In addition the program offers health and life-skill education, case management, effective utilization of community resources, and job readiness/education attainment to participants during and after their pregnancy. BLUES is currently running in two of the most affected areas in Tennessee; Memphis in Shelby County, and Chattanooga in Hamilton County.

In partnership with the BLUES Team, the Office of Biomedical Informatics at UTHSC has developed an online registry and subject tracking and management application within the Slim-Prim integrated data system to serve as a central, web-accessible subject data management system. Core functionalities include intuitive screening tools, participation registry, graphical calendar for tracking and event planning, individual and group session scheduling, personalized task reminder, and report generator. The system is designed for simplicity, and uses standardized questions to evaluate health outcomes. Incoming data sets are monitored by the biomedical informatics and research teams. Personalized task reminders are auto-generated to coordinate social workers and nurse coordinators to follow up on their assigned participants. Reports are regularly created for close communication among the research teams.

## Results

This system has already facilitated the funding of expansion grant applications. Long term plans include instantiation of the system to serve as a nationally available resource for similar health disparities projects.

